# Double Intussusceptions in a 20-Year-Old Lady Harboring a Tubulovillous Adenoma with High-Grade Dysplasia: A Case Report and Literature Review

**DOI:** 10.7759/cureus.34265

**Published:** 2023-01-27

**Authors:** Fawaz Alkeraithe, Ahmad Alghamdi, Abdullah Algharbi, Anas Aluwishiq, Zeyad Alsolami

**Affiliations:** 1 Department of Urology, King Fahad Medical City, Riyadh, SAU; 2 Department of General Surgery, King Fahad Medical City, Riyadh, SAU

**Keywords:** double, dysplasia, colonic adenoma, tubulovillous, intussusception

## Abstract

Underlying malignancy is a concern when intussusception is diagnosed in adults and the elderly. Management includes oncological resection of the intussusception. We report a case of a 20-year-old female patient who presented with signs of bowel obstruction. Computed tomography demonstrated double intussusceptions (ileo-cecal and transverse colo-colonic). During laparotomy, the mid-transverse intussusception reduced spontaneously while the other did not. Both intussusceptions were managed with oncological resection. The final pathology showed a tubulovillous adenoma with high-grade dysplasia. As a result, intussusception in adults should be investigated thoroughly to exclude malignant potential.

## Introduction

Intussusception is the invagination of an intestinal segment into an adjacent proximal or distal one [1]. Intussusception is common in children but instead is far less common in adults and usually has a leading point which may indicate malignancy in > 50% of cases [2]. Therefore, computed tomography (CT) is used as the medical imaging of choice [1]. In adults, surgical resection of intussusception following oncological rules is the treatment of choice because of the possibility of malignancy. This paper reports an uncommon finding in a 20-year-old female patient with ileo-cecal and transverse colo-colonic double intussusceptions, treated by oncological surgical resection.

## Case presentation

A 20-year-old female patient presented to the emergency department (ED) at a tertiary center with a one-week history of epigastric pain, nausea, and vomiting. She could not tolerate oral intake. The pain was associated with one episode of passing bloody stool with clots five days before ED presentation. After that, she experienced constipation. She mentioned occasional constipation for the last three months. She denied fever at home or a family history of colorectal cancer.

The patient's vital status in the ED showed a blood pressure of 122/62 mmHg and a pulse of 84 beats/minute. Upon physical examination, the patient was conscious, alert, and oriented. She did not show any sign of distress. She had a palpable tender epigastric mass with a soft abdomen and no signs of peritonitis. Rectal examination revealed a stain of stool without blood.

**Figure 1:**
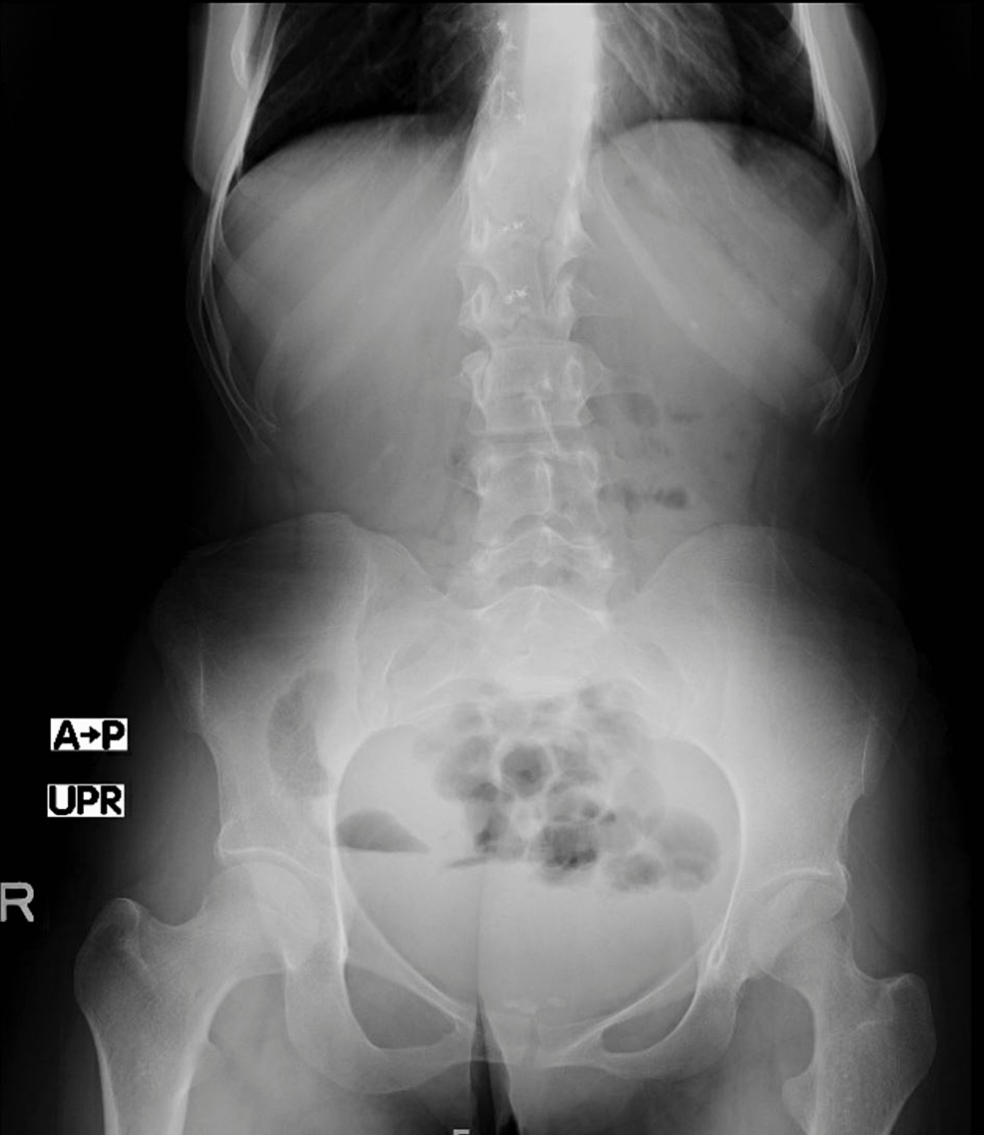
Abdominal X-ray showed no signs of air-fluid level or pneumoperitoneum

**Figure 2:**
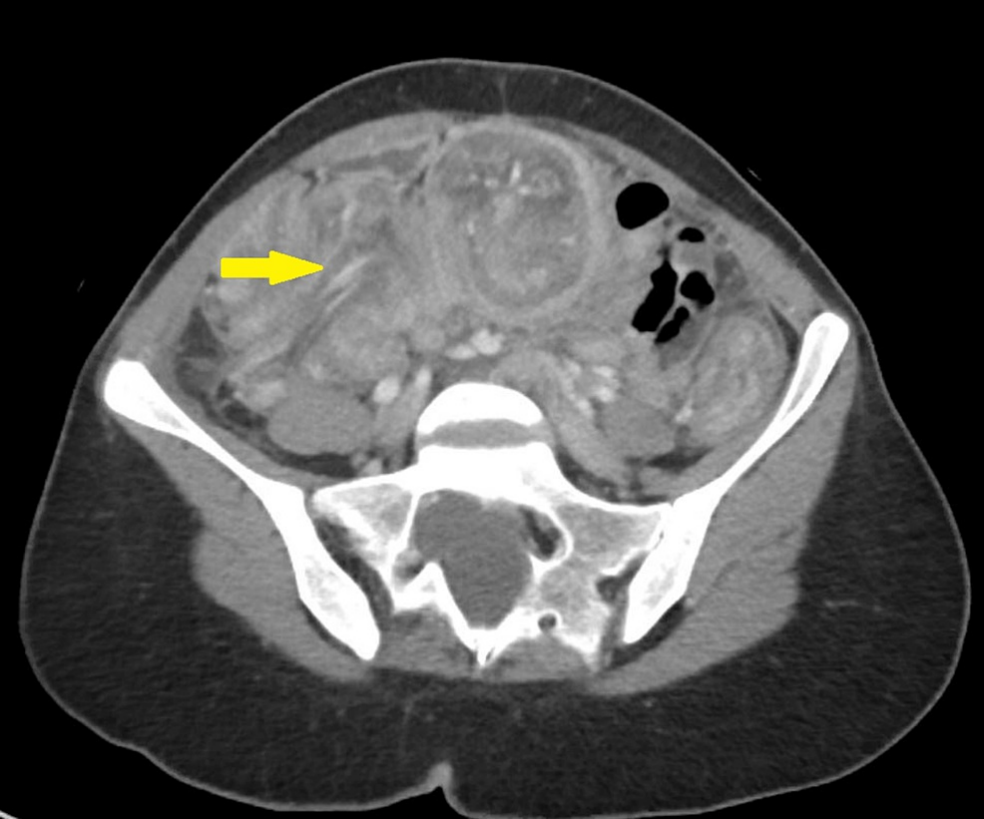
Cecal-colonic intussusception in the axial cut of abdominal computed tomography scan (yellow arrow)

**Figure 3:**
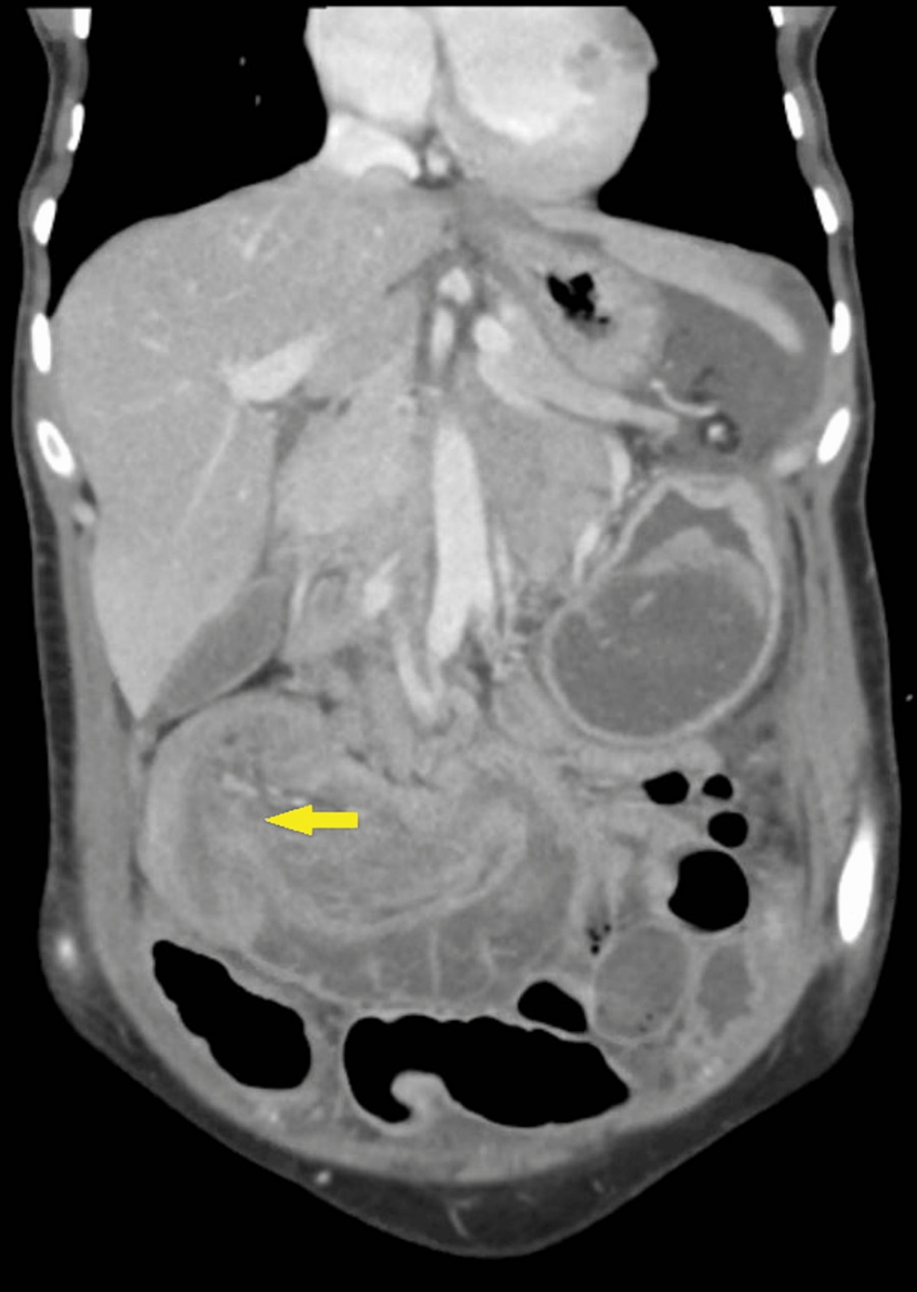
Cecal-colonic intussusception in the coronal cut of abdominal computed tomography scan (yellow arrow)

**Figure 4:**
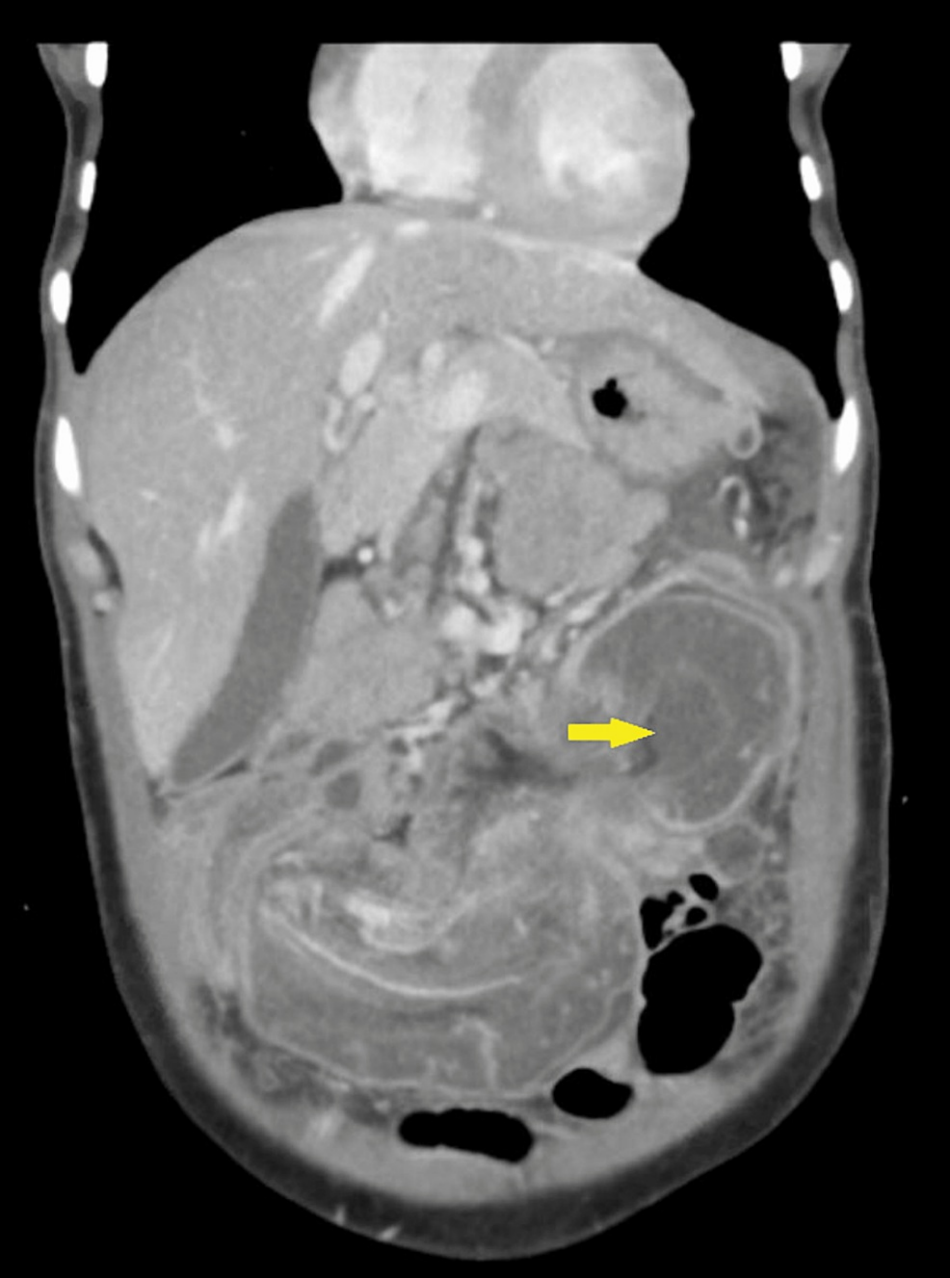
Transverse colo-colonic intussusception in the coronal cut of abdominal computed tomography scan showing “sausage sign” (yellow arrow)

**Figure 5:**
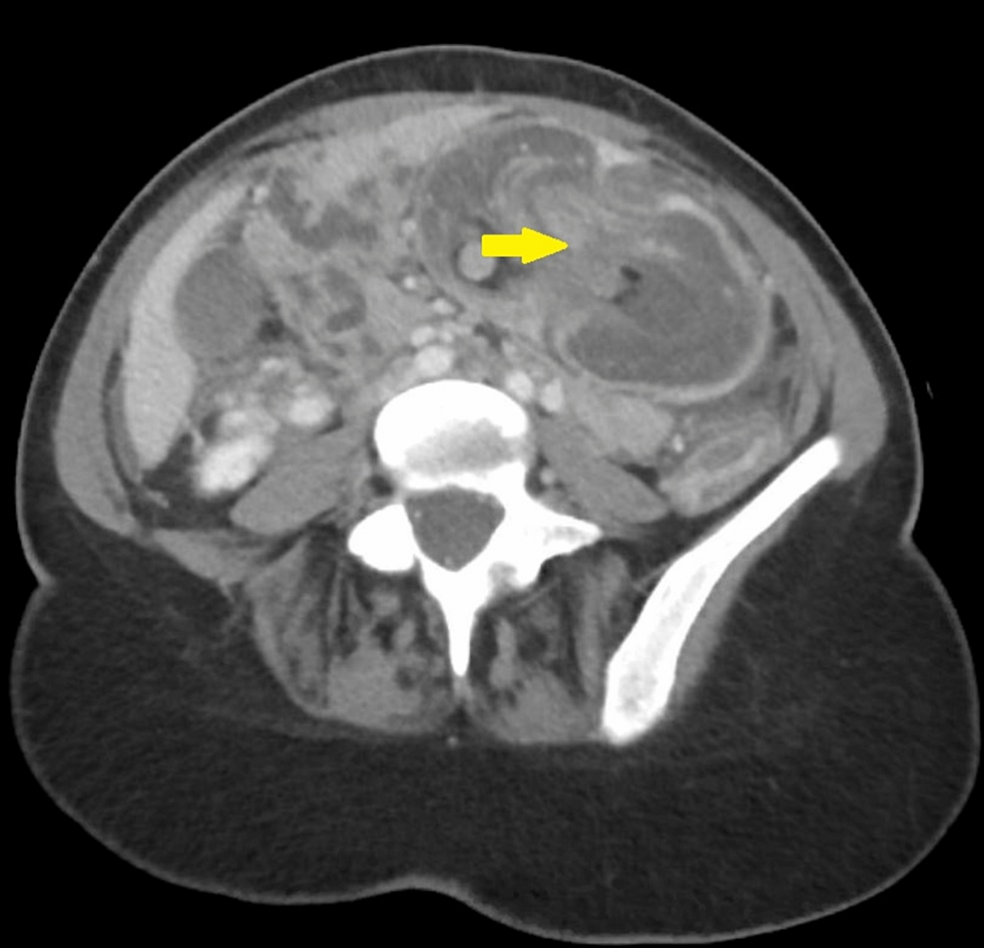
Transverse colo-colonic intussusception in the axial cut of abdominal computed tomography scan (yellow arrow)

Lab tests were as follows: white blood cell count, 10.6 × 103/uL; hemoglobin, 13.8 g/dL; serum sodium, 139 mmol/L; serum potassium, 3.23 mmol/L; negative polymerase chain reaction clostridium test; lipase, 108 U/L; amylase, 142 U/L; and venous lactate, 1.0 mmol/L. Chest and abdomen X-rays were unremarkable (Figure 1). CT on the abdomen showed two areas of colo-colonic intussusception in the ileo-cecal and mid-transverse colon (Figures 2, 3, 4, 5). Notably, the intussusceptions were associated with partial small bowel obstruction and dilated small bowel loops reaching 35 mm. Moreover, diffuse edematous changes were seen in the distal ileum and descending colon.

**Figure 6:**
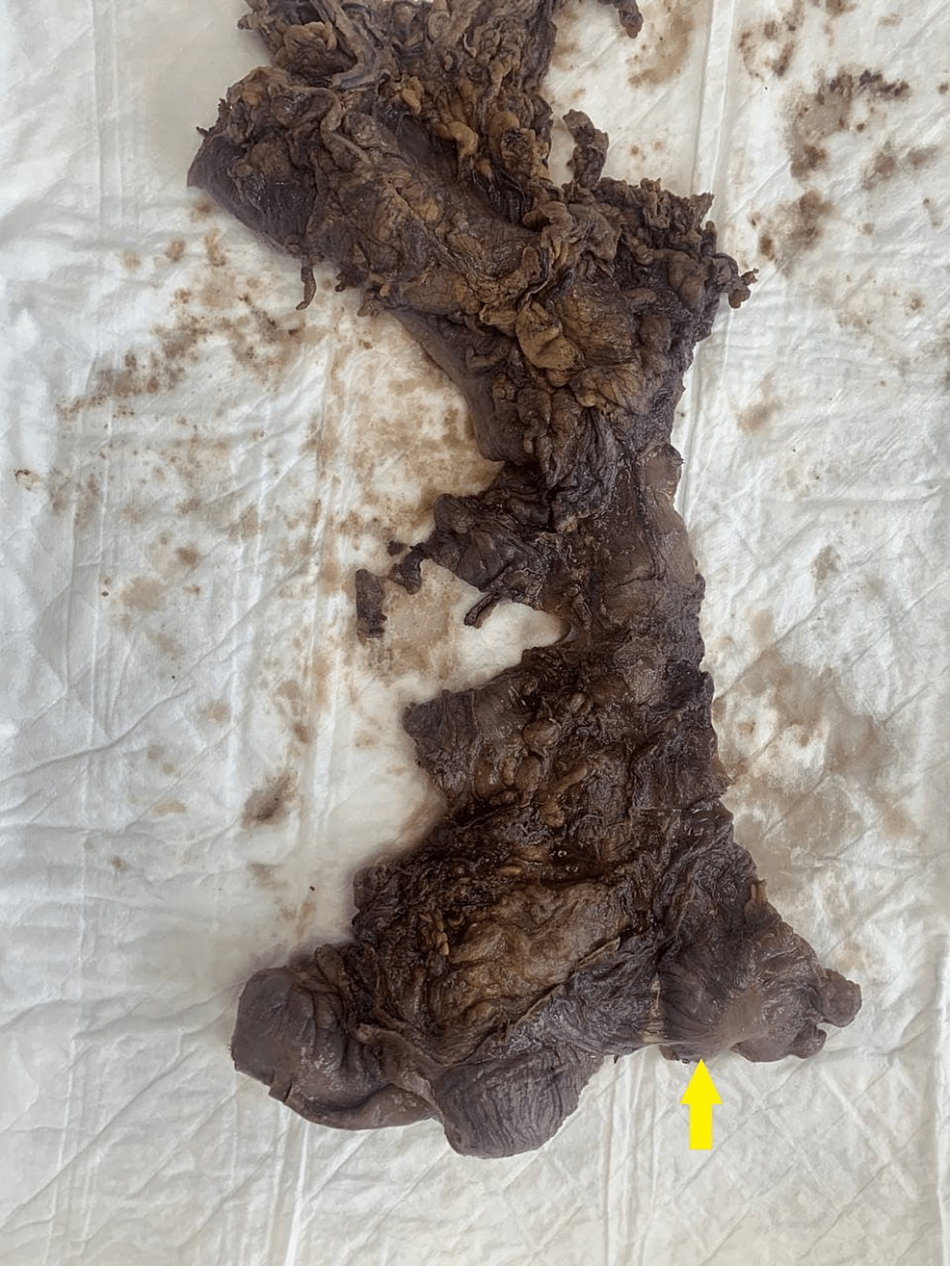
View of the surgical specimen showing thickened cecum

Upon laparotomy, two areas of intussusceptions were present, as described earlier. The transverse colo-colonic intussusception reduced spontaneously, while the ileo-cecal one reduced after manipulation and resection. The small bowel looked healthy without obvious pathology. The cecum was thickened without other pathological findings (Figure 6). We proceeded with oncological resection with extended right hemicolectomy and side-to-side ileo-transverse anastomosis. The postoperative period was unremarkable. She tolerated a soft diet and passed stool. The final pathology turned out to be tubulovillous adenoma with high-grade dysplasia, and the patient was referred to a gastroenterologist for colonoscopy. 

## Discussion

A 20-year-old female with no family history of cancers presented with colonic adenomas, which are rarely encountered in adults. She complained of partial intestinal obstruction due to double intussusceptions. Intussusception is a surgical emergency that may occur at any age with different etiologies. In adults and the elderly, underlying malignancy is more likely present in contrast to the pediatric age group, where it is commonly idiopathic. It rarely occurs in adolescents and young adults, though it is associated with many autoimmune vasculitides [3]. In this case, the patient presented in early adulthood with high-grade adenoma. This unusual presentation is commonly seen in adults and should be ruled out in the elderly with a high risk for adenomas and adenocarcinomas of the colon and malignancy [4].

The classical clinical manifestations of intussusception are intestinal obstruction where symptoms usually include abdominal pain that is colicky in nature, constipation with passing jelly mucoid secretion, and visible peristaltic wave with a palpable abdominal mass [5]. This presentation is not typical in adults. Our patient presented with symptoms of partial intestinal obstruction, including oral intake intolerance associated with nausea, vomiting, and epigastric pain. Passing a blood clot is a typical presentation of adulthood colorectal neoplasm [3]. The standard imaging technique for adults presenting with intussusception is a multi-slice abdominal-pelvic CT scan with contrast materials to demonstrate the presence of neoplastic mass. Double intussusception with double cancer locations (ileo-cecal and transverse colo-colonic) is uncommon in sporadic colorectal cancer or colorectal adenoma [3]. Contrastingly, in hereditary colonic neoplasms, such as familial polyposis coli and hereditary nonpolyposis coli, double intussusceptions may occur due to the presence of multiple foci of adenomas and adenocarcinoma. Genetic and molecular screening should be planned for this patient even if there is no family history of cancer. This is because the genetic abnormality may be harbored in the germ line cells. Follow-up and prophylactics should be considered in these cases [3]. Surgical management in pediatric patients focuses on bowel preservation with nonoperative reduction of the intussusception and decreasing operative interventions. This is due to the low risk of malignancy in these population groups [6]. Operative reduction can be considered in older patients who have a low risk for malignancy. Though our patient is young and had no risk of developing colonic cancer, the intraoperative finding showed a thickened cecum wall pointing toward the presence of a neoplastic process. This necessitated bowel resection by right hemicolectomy and ileocolonic anastomosis. Fortunately, histopathological examination reported the presence of noninvasive dysplastic tubulovillous adenoma with no polyposis syndrome. Therefore, no further management was needed other than colonic follow-up for the next year after surgical excision [6].

## Conclusions

This case report shows an uncommon presentation of double intussusceptions in a young adult patient. It highlights the importance of a comprehensive assessment of adult intussusception and follow-up of the oncological resection for possible malignancy. It can highly be a malignant precursor which requires follow-up as in our case.
